# Self-Illuminating Agents for Deep-Tissue Optical Imaging

**DOI:** 10.3389/fbioe.2019.00326

**Published:** 2019-11-12

**Authors:** Qing Li, Jianfeng Zeng, Qingqing Miao, Mingyuan Gao

**Affiliations:** State Key Laboratory of Radiation Medicine and Protection, School for Radiological and Interdisciplinary Sciences (RAD-X), Collaborative Innovation Center of Radiation Medicine of Jiangsu Higher Education Institutions, Soochow University, Suzhou, China

**Keywords:** self-luminescence, bioluminescence, chemiluminescence, afterglow, deep-tissue imaging, optical imaging

## Abstract

Optical imaging plays an indispensable role in biology and medicine attributing to its noninvasiveness, high spatiotemporal resolution, and high sensitivity. However, as a conventional optical imaging modality, fluorescence imaging confronts issues of shallow imaging depth due to the need for real-time light excitation which produces tissue autofluorescence. By contrast, self-luminescence imaging eliminates the concurrent light excitation, permitting deeper imaging depth and higher signal-to-background ratio (SBR), which has attracted growing attention. Herein, this review summarizes the progress on the development of near-infrared (NIR) emitting self-luminescence agents in deep-tissue optical imaging with highlighting the design principles including molecular- and nano-engineering approaches. Finally, it discusses current challenges and guidelines to develop more effective self-illuminating agents for biomedical diagnosis and treatment.

## Introduction

Imaging techniques such as single-photon emission computed tomography (SPECT), positron emission computed tomography (PET), magnetic resonance imaging (MRI), computed tomography (CT), optical imaging, ultrasound imaging, and photoacoustic (PA) imaging have become powerful tools to detect and monitor the physiological or pathological processes at the molecular, subcellular, cellular, tissue and body levels (Jokerst and Gambhir, 2011; Farzin et al., 2018; Li et al., 2018; Ma et al., 2018; Ni et al., 2018a; Yin et al., 2019). Among the above-mentioned imaging modalities, optical imaging shows tremendous potential in biomedical applications due to its noninvasiveness, high sensitivity, high temporal and spatial resolution (Liu et al., 2007; Weissleder and Pittet, 2008; Choi et al., 2013; Zhu et al., 2018). Moreover, optical imaging equipment are relatively low-cost and convenient to operate (Baker, 2010; Badr and Tannous, 2011; Gnaim et al., 2019). However, as a conventional optical imaging, fluorescence imaging has not been extensively utilized in clinical practice. The main reason is the need for concurrent light excitation in imaging process, which produces severe light-tissue interactions (i.e., light scattering, tissue absorption, and autofluorescence), consequently resulting in poor signal-to-background ratio (SBR) and low penetration depth (Shimon et al., 2009; Jones et al., 2017; Miao and Pu, 2018).

**Scheme 1:**
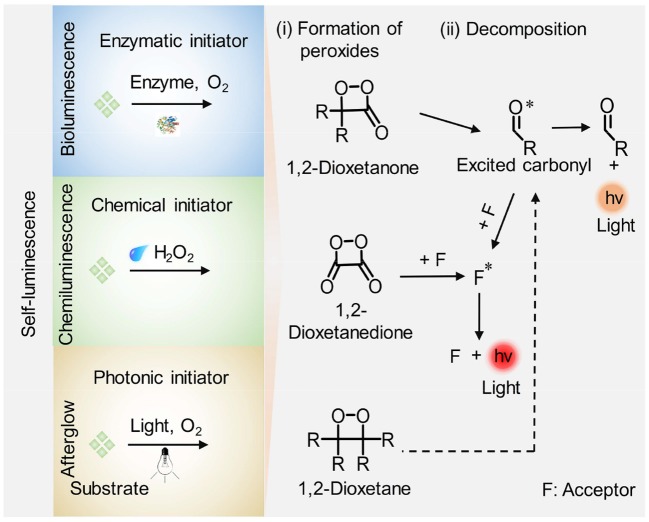
Schematic illustration of the general mechanism for generation of self-luminescence including bioluminescence, chemiluminescence, and afterglow luminescence. (i) Formation of high-energy peroxides: enzymes, reactive species (i.e., H_2_O_2_), and light irradiation facilitate the formation of high-energy intermediates (cyclic four-membered peroxides such as 1,2-dioxetanone, 1,2-dioxetanedione, and 1,2-dioxetane) in bioluminescence, chemiluminescence, and afterglow luminescence imaging process, respectively. (ii) A chemiexcitation process: the peroxides decompose directly or activated by an acceptor (F) through energy transfer, leading to the formation of an excited state (excited carbonyl or F^*^) accompanied by light emission.

Self-luminescence imaging, which does not rely on real-time light excitation and thus eliminates the tissue autofluorescence and photo-bleaching, has attracted increasing attention in recent years. By virtue of the merits, self-luminescence imaging displays higher imaging sensitivity, higher SBR, and deeper imaging depth relative to fluorescence imaging (Table 1; Chen et al., 2017; Hananya and Shabat, 2017; Yan et al., 2019). To date, three kinds of self-luminescence imaging approaches including bioluminescence, chemiluminescence and afterglow luminescence have been developed and widely applied in biology and medicine. Among the self-luminescence imaging techniques, bioluminescence and chemiluminescence imaging do not need external light source and detect photons from enzymatic- and reactive species-initiated oxidation reaction with their substrates, respectively. By contrast, instead of the combination of enzyme/reactive species with corresponding substrate to generate photons, afterglow luminescence imaging necessitates a pre-irradiation of light to store the energy in agents and then collects the slowly releasing photons from the stored energy after the cessation of light irradiation. The underlying process to generate self-luminescence is summarized in a general way Scheme 1. Bioluminescence, chemiluminescence, and afterglow luminescence rely on respective initiator (enzymes for bioluminescence, H_2_O_2_ for chemiluminescence, and light irradiation for afterglow luminescence) to generate high-energy peroxides such as 1,2-dioxetanone, 1,2-dioxetanedione, and 1,2-dioxetane firstly. Then the peroxides dissociate directly or modulating by an acceptor molecule (F) through energy transfer, leading to the production of an excited state (an excited carbonyl or acceptor molecule) and subsequent light emission.

**Table 1:** Comparison of the penetration depth and SBR for self-luminescence imaging modalities.

**Imaging modality**	**Penetration depth (cm)**	**SBR**	**References**
Bioluminescence	2	100	Xiong et al., 2012
Chemiluminescence	2.5	N.A.	Shuhendler et al., 2014
Afterglow	5	2,922	Jiang et al., 2019

*N.A., Not applicable*.

Near-infrared (NIR) self-luminescence imaging has attracted increasing enthusiasm due to higher tissue penetration depth of NIR light than visible light, which dramatically expands the visualization scope of physiological or pathological processes in living subjects in a noninvasive way (Hasegawa et al., 2013; Li et al., 2016; Zhen et al., 2017). The publications and citations regarding NIR self-illuminating agents are increasing in number, and there are few reviews summarizing the recent development and advances of NIR self-illuminating agents for biomedical applications (Hananya and Shabat, 2017; Weihs and Dacres, 2019). This review will focus on the recent progress on NIR emitting self-luminescence agents for deep-tissue optical imaging, and pinpoint their contemporary molecular- and nano-engineering approaches that have been exploited in this field. As follows, the molecular construction and applications of NIR bioluminescence imaging are first described. Then, NIR chemiluminescence imaging and afterglow luminescence imaging are discussed, respectively, highlighting the strategies for red-shifting and amplifying the luminescence. Finally, it discusses current challenges and guidelines to develop more effective self-illuminating agents for biomedical diagnosis and treatment.

## NIR Bioluminescence Imaging

Bioluminescence is the occurrence of light emission generated through oxidation reaction of a substrate catalyzed by an enzyme. The typical enzyme that is used for bioluminescence imaging is luciferase including firefly luciferase (Fluc), *Gaussia* luciferase (Gluc), *Renilla* luciferase (Rluc), and Nanoluc (Nluc) (Kaskova et al., 2016). Nevertheless, the naturally occurring bioluminescence is commonly resided in the visible region (Hai et al., 2017; Yao et al., 2018; Zhang et al., 2018). For example, the native substrate for Fluc is D-luciferin with an emission peak at 560–610 nm, the native substrate for Gluc and Rluc is coelenterazine with an emission maximum at 480 nm, and the native substrate for Nluc is furimazine with an emission peak at 460 nm (Hall et al., 2012; Tang et al., 2019b). As a result, the bioluminescence in the visible region suffers from severe tissue attenuation, compromising imaging SBR and imaging depth, which is not appropriate for *in vivo* deep-tissue imaging. To resolve this, some approaches were adopted to red-shift the light from visible into the NIR region (650–950 nm) to achieve higher imaging depth due to the decreased light scattering and tissue absorption of NIR photons through living tissues relative to that of visible light (Mezzanotte et al., 2017).

**Figure 1:**
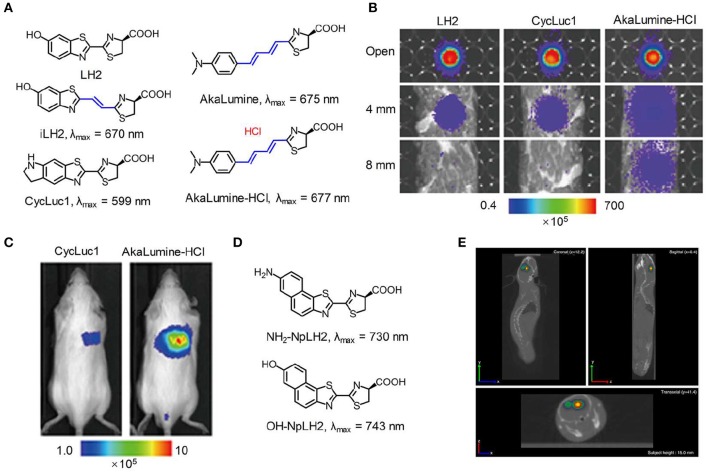
NIR bioluminescence imaging. **(A)** Chemical structures of LH_2_, iLH_2_, CycLuc1, AkaLumine, and AkaLumine-HCl. **(B)** Bioluminescence imaging of LH_2_, CycLuc1, or AkaLumine-HCl through biological tissues with different thickness (0, 4, and 8 mm). The penetration efficiency was calculated according to the relative bioluminescence imaging intensities. **(C)** Representative bioluminescence imaging of lung metastasis in living mice after intraperitoneal injection of substrates (5 mM, 100 μL) (reproduced with permission from Kuchimaru et al., 2016). **(D)** Chemical structures of NH_2_-NpLH_2_ and OH-NpLH_2_. **(E)** Co-registered CT and bioluminescence tomography imaging of mice brain using the bioluminescence agent CBR2opt/NH2-NpLH_2_. Migration of cells could be clearly observed at day 5 after cell transplantation (reproduced with permission from Hall et al., 2018).

A general strategy for constructing NIR emitting bioluminescence is to elongate the π-conjugation of luciferase substrate (Miura et al., 2013). To explore the substrate with an emission wavelength in the NIR region, Pule et al. synthesized a luciferin analog (iLH_2_) by inserting a carbon-carbon double bond between the thiazole group and benzothiazole group of luciferin (LH_2_). The substrate iLH_2_ produced light in the NIR range (λ_max_ = 670 nm) in presence of a native Fluc (Figure 1A) and showed an enhanced penetration depth through blood relative to luciferin (Jathoul et al., 2014). The *in vivo* imaging ability of iLH_2_ was investigated by establishing different tumor models in mice. The results revealed that the iLH_2_ had less tissue attenuation and showed more imaging definition for systemic lymphoma and metastatic tumor in mice compared to LH_2_. Using a similar strategy, Iwano et al. synthesized another luciferin analog, Akalumine, and utilized it for tumor imaging (λ_max_ = 675 nm) (Iwano et al., 2013). However, Akalumine had poor water solubility, hampering its efficient accumulation to the targeted site. To resolve this, the same group reported a substituent, AkaLumine hydrochloride (AkaLumine-HCl), which had better water-solubility and emitted NIR light (λ_max_ = 677 nm) in the presence of luciferase (Kuchimaru et al., 2016). After penetrating 4- or 8-mm-thick tissue, the bioluminescense intensity of AkaLumine-HCl was 5- and 8.3-fold higher than that of D-luciferin, and 3.7- and 6.7-fold higher than that of CycLuc1, respectively (Figure 1B). As expected, Akalumine-HCl performed imaging of lung metastases with higher sensitivity relative to CycLuc1 (Figure 1C). To further red-shift the bioluminescence, Mezzanotte developed two kinds of naphthyl-based luciferin analogs (i.e., NH_2_-NpLH_2_ and OH-NpLH_2_) that were matched with a mutant luciferase (CBR2), generating NIR bioluminescence (730 nm for NH_2_-NpLH_2_ and 743 nm for OH-NpLH_2_) (Hall et al., 2018; Figure 1D). In addition, the mutant enzyme/substrate (CBR2opt/NH_2_-NpLH_2_) system enabled stable and highly resolved NIR bioluminescence imaging of the migration of cells in the brain (Figure 1E).

**Figure 2:**
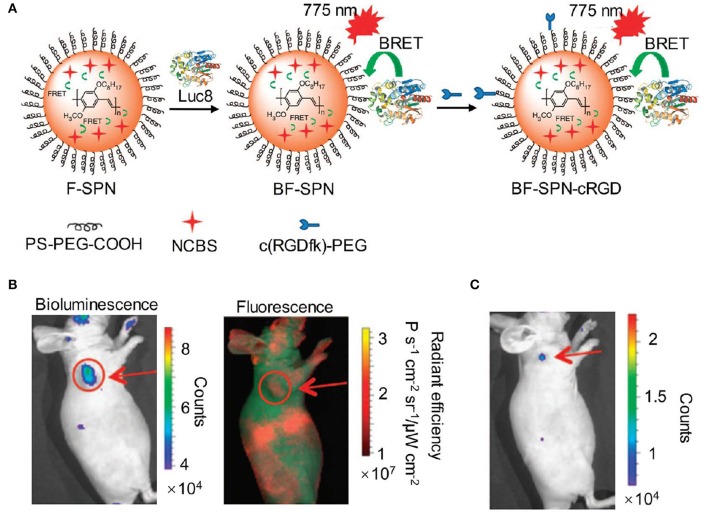
NIR bioluminescence imaging. **(A)** Illustration of the fabrication of NIR self-luminescence nanoparticle BF-SPN-cRGD by a BRET-FRET strategy. **(B)** Bioluminescence (left) and fluorescence (right) imaging of U87MG tumor in living mice at *t* = 5 min post-injection of BF-SPN-cRGD. **(C)** Bioluminescence imaging of U87MG tumor (2 mm) at *t* = 2 h post-injection of BF-SPN-cRGD (reproduced with permission from Xiong et al., 2012).

To avoid the time-consuming synthetic work, bioluminescence resonance energy transfer (BRET) is an alternative approach to red-shift the light emission from visible into the NIR region. To achieve this, a NIR-emitting dye or nanoparticle was commonly selected to covalently conjugate with the natural bioluminescence system as an acceptor to allow for efficient energy transfer (Weihs and Dacres, 2019). Rao et al. developed a series of BRET-based NIR bioluminescence inorganic systems, in which a *Renilla reniformis* luciferase (Luc8) served as an energy donor and quantum dots (QDs) acted as an energy acceptor (So et al., 2006a,b; Yao et al., 2007; Ma et al., 2010). Such QDs-based BRET systems engineering with emissions ranging from visible to the NIR were utilized for *in vivo* imaging and enzyme activity detection with high sensitivity. Except for inorganic BRET systems, Rao et al. also tried to introduce organic systems for constructing BRET-based NIR bioluminescence. For instance, self-luminescent semiconducting polymer nanoparticles (BF-SPN) were developed for *in vivo* NIR bioluminescence imaging by combining BRET and fluorescence resonance energy transfer (FRET) (Xiong et al., 2012). The nanoparticles (BF-SPNs) were designed to comprise three components: Luc8, poly [2-methoxy-5-((2-ethylhexyl)oxy)-p-phenylenevinylene] (MEHPPV) and silicon 2,3-naphthalocyanine bis(trihexylsilyloxide) (NCBS) serving as the BRET donor, the BRET acceptor (and the FRET donor) and the FRET acceptor, respectively. Such BRET-FRET system produced multiple energy transfer from Luc8 to MEHPPV and then to NCBS, resulting in final NIR bioluminescence centered at 780 nm. In order to improve the targeted ability toward tumor, the cyclic arginine-glycine-aspartic (cRGD) peptides were attached to the surface of SPN to obtain BF-SPN-cRGD (Figure 2A). As shown in Figure 2B, the tumor could be clearly delineated by BF-SPN-cRGD with bioluminescence imaging while it could not be achieved with NIR fluorescence imaging, attributing to negligible background of bioluminescence in living tissue. In addition, the bioluminescence imaging of BF-SPN-cRGD could detect smaller tumors (2–3 mm in diameter) with SBR over 100, which was 30-fold higher than that of fluorescence imaging (Figure 2C).

## NIR Chemiluminescence Imaging

**Figure 3:**
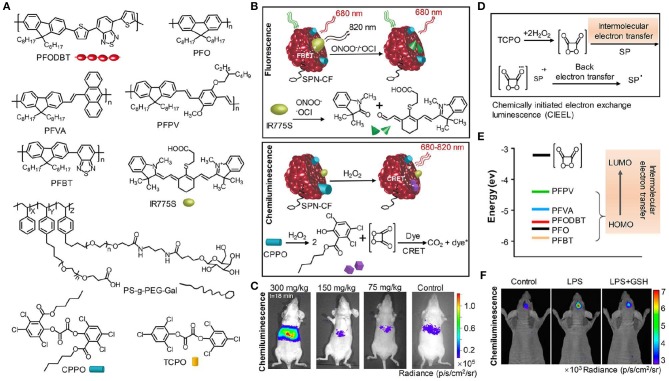
NIR chemiluminescence imaging. **(A)** Chemical structures of PFODBT, PFO, PFVA, PFPV, PFBT, IR775S, PS-g-PEG-Gal, CPPO, and TCPO. **(B)** Illustration of the detection mechanism for H_2_O_2_ and ONOO^−^ or ^−^OCl using NIR chemiluminescence probe SPN-CF. **(C)** Representative chemiluminescence images of mice after treatment with different APAP dosages, from left to right (300, 150, 75 mg/kg, or saline), followed by injection of 0.8 mg SPN-CF (reproduced with permission from Shuhendler et al., 2014). **(D)** Schematic for the mechanism of CIEEL from TCPO to SPNs. **(E)** The HOMO levels of SPs and the LUMO level of 1,2-dioxetanedione. **(F)** Representative chemiluminescence imaging of mice brain after treatment with saline, LPS (10 mg/mL, 2 μL) or LPS with GSH (500 mg/mL, 1 μL), followed by an intracerebral injection of SPN-PFPV (10 mg/mL, 2 μL) at *t* = 4 h later (reproduced with permission from Zhen et al., 2016).

Compared with bioluminescence imaging, chemiluminescence imaging does not need an enzyme and detects light emission from the reaction of a substrate with reactive oxygen species (ROS), which performs a great deal of versatility and flexibility in experimental systems (Augusto et al., 2013; Ryan and Lippert, 2018; Vacher et al., 2018). The reaction commonly contains two sequential processes: oxidation of a substrate by ROS leads to the formation of a high energy intermediate and subsequently the high energy intermediate decomposes along with the generation of light. Chemiluminescence assays have been widely used in various biological and chemical applications due to their excellent sensitivity and high SBR (Suzuki and Nagai, 2017; Sun et al., 2018; Tiwari and Dhoble, 2018; Son et al., 2019). These advantages accelerate the development of novel chemiluminescent probes. To obtain NIR chemiluminescence which is more suitable for *in vivo* imaging (Green et al., 2017a; Lippert, 2017), strategies including chemiluminescence resonance energy transfer (CRET) and chemical modifications of the chemiluminescent substrate have been utilized to achieve bright and NIR chemiluminescence probes, enabling deep-tissue imaging (Lim et al., 2010; Hananya and Shabat, 2019; Nishihara et al., 2019). Oxalate esters and luminol are the typical H_2_O_2_-activated chemiluminescent compounds, which have been applied to visualize H_2_O_2_-related biological situations. To improve deep-tissue imaging ability of oxalate esters and luminol, the CRET is a facile way to shift their visible chemiluminescence into the NIR region. For instance, Rao et al. exploited an organic semiconducting polymer nanoparticle-based NIR chemiluminescence probe (SPN-CF) by integrating CRET and FRET for detecting peroxynitrite (ONOO^−^) and hydrogen peroxide (H_2_O_2_) (Shuhendler et al., 2014). In SPN-CF, bis-(2,4,5-trichloro-6-(pentyloxycarbonyl)phenyl) oxalate (CPPO) served as the chemiluminescent substrate for reaction with H_2_O_2_ and poly(2,7-(9,9′-dioctylfluorene)-alt-4,7-bis(thiophen-2-yl)benzo-2,1,3-thiadiazole) (PFODBT, 680 nm) acted as both the CRET acceptor and the FRET donor. A cyanine dye (IR775S, 820 nm) was embedded into the nanoparticles to serve as the FRET acceptor, inducing the NIR chemiluminescence. SPN-CF was anchored with galactose-attached copolymer (PS-g-PEG-Gal) favoring hepatocytes-targeting (Hu et al., 2013; Figure 3A). *In vitro* experiment confirmed that the SPN-CF could detect H_2_O_2_ and ONOO^−^ with a limit of detection (LOD) of 5 and 10 nM, respectively (Figure 3B). The SPN-CF was further explored to detect hepatotoxicity induced by the anti-pyretic acetaminophen (APAP) (McGill and Jaeschke, 2013). Dose-dependent ROS/reactive nitrogen species (RNS) in the liver could be detected by the SPN-CF within minutes of APAP challenge (Figure 3C), antedating histological changes in drug-damaged tissue. This study proved that the SPN-CF could be developed for early detection of hepatotoxicity *in vivo*.

To further enhance the chemiluminescence efficacy, Pu group reported the screening of five polyfluorene derivatives-based luminescent reporters (PFO, PFVA, PFPV, PFPT, and PFODBT) to pair with the substrate bis(2,4,6-trichlorophenyl) oxalate (TCPO) (Zhen et al., 2016), as shown in Figure 3A. Among all the SPNs, the highest occupied molecular orbital (HOMO) of PFPV was closest to the lowest unoccupied molecular orbital (LUMO) of 1,2-dioxetanedione (Figure 3E), which led to the fastest intermolecular electron transfer between them and thereby created the highest chemiluminescence intensity based on the chemically initiated electron exchange luminescence (CIEEL) mechanism (Figure 3D). The optimized system (SPN-PFPV) showed a quantum yield (QY) of 2.3 × 10^−2^ einsteins/mol, superior to the previously reported probes (Lim et al., 2010; Lee et al., 2012; Augusto et al., 2013). Furthermore, the PFPV-SPN had a high sensitivity to detect H_2_O_2_ with LOD as low as 5 nM. To endow this probe with NIR chemiluminescence for *in vivo* imaging, a NIR dye (NCBS) as an energy acceptor was doped into SPN-PFPV to obtain SPN-PFPV-NIR with 778 nm emission by CRET. The SPN-PFPV-NIR allowed the ultrasensitive detection of H_2_O_2_ *in vivo* in both peritonitis and neuroinflammation models (Figure 3F).

Recently, the NIR self-illuminating nanomaterials based on luminol system were developed for inflammation imaging (Liu et al., 2019; Xu et al., 2019). Zhang synthesized a biodegradable polymer nanoparticle conjugating chlorin e6 (Ce6) and luminol (CLP) (Xu et al., 2019). In the presence of myeloperoxidase (MPO) and ROS, luminol emitted luminescence at 440 nm, which was shifted to the Ce6 emission (675 nm) via energy transfer. The ROS-induced oxidation of luminol and efficient energy transfer endowed the self-illuminating CLP nanoparticle with an outstanding *in vivo* imaging capability to detect inflammation in various animal models.

Different from above-mentioned chemiluminescent compounds which require both sequential oxidation and decomposition steps in imaging process, dioxetane-based chemiluminescent agent is an oxidized high-energy species, which eliminates the ROS-oxidation step and directly generates light emission after substrate decomposition. The dioxetane can remain stable at room temperature until phenol-protecting group was removed to initiate the decomposing process, which is an ideal scaffold for designing stimulus-responsive chemiluminescent probes. To date, dioxetane-based chemiluminescent probes have been well developed for detecting enzymes and other analytes (Cao et al., 2016; Ryan and Lippert, 2018; Sun et al., 2018; Hananya and Shabat, 2019). To construct NIR dioxetane agents, Shabat et al. recently reported a “turn-on” NIR fluorophore-tethered dioxetane chemiluminescence probe for β-galactosidase imaging (Hananya et al., 2016). In the presence β-galactosidase, the protecting group of dioxetane as the CRET donor was removed to form the electronically excited benzoate and then the energy was transferred to NIR fluorophore (QCy) acting as the CRET acceptor, which resulted in efficient and bright NIR chemiluminescence (714 nm). The chemiluminescence intensity of the probe was enhanced by 100-fold compared to the probe without tethering NIR fluorophore. Moreover, after incubation with β-galactosidase, the NIR fluorophore-tethered dioxetane probe was then injected into living mice and obtained clear chemiluminescence imaging with 6-fold increase in signal intensity than that obtained by green fluorophore-tethered dioxetane probe.

**Figure 4:**
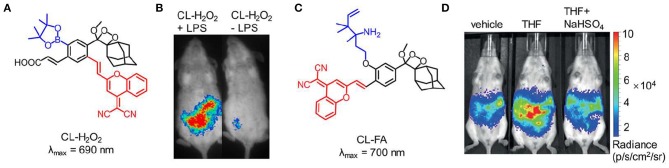
NIR chemiluminescence imaging. **(A)** Chemical structure of probe CL-H_2_O_2_. **(B)** Representative chemiluminescence images of living mice treated with LPS (0.1 mg/mL, 1 mL) (left) or PBS buffer (1 mL, pH 7.4) (right), followed by *t* = 4 h post-injection of probe CL-H_2_O_2_ (reproduced with permission from Green et al., 2017b). **(C)** Chemical structure of probe CL-FA. **(D)** Representative chemiluminescence imaging of endogenous FA produced through tetrahydrofolate (THF) metabolism (reproduced with permission from Bruemmer et al., 2018).

Although the NIR chemiluminescent probes based on dioxetane can be easily obtained by CRET, the chemiluminescence intensity is limited by the energy-transfer efficiency. An alternative strategy is to develop NIR chemiluminescence probe with a direct emission mode through structural modification. Recently, Shabat et al. put forward a design strategy to generate NIR chemiluminescence by extending π-conjugation length of the chemical substrate. Based on the design, a NIR-emitting phenoxy-dioxetane luminophore (690 nm) with dicyanomethylene-4H-chromene (DCMC) as an electron acceptor was synthesized (Green et al., 2017b). To achieve the ability to detect H_2_O_2_, the phenol group of this luminophore was masked with aryl-boronate that can react with H_2_O_2_ to generate the active phenolate-dioxetane (Figure 4A). This probe was investigated to monitor H_2_O_2_ in living mice and the inflammation induced by lipopolysaccharide (LPS). Figure 4B shows that the inflammation for LPS-treated mice was clearly visualized with stronger chemiluminescence signal compared with that of non-LPS-treated mice. In 2018, another NIR-emitting phenoxy-dioxetane luminophore with a 2-aza-Cope FA-reactive trigger was reported for monitoring formaldehyde (FA) (Figure 4C; Bruemmer et al., 2018). With NIR chemiluminescence at 700 nm, the luminophore was evaluated for *in vivo* FA imaging in mice, which confirmed that this probe could detect clearly endogenous FA released from the folate metabolism (Figure 4D).

## NIR Afterglow Imaging

Bioluminescence and chemiluminescence require respective enzyme or ROS to oxidize their substrate to generate luminescence, and thus the imaging signal was easily disturbed by cellular environment and substrate availability. Different from bioluminescence and chemiluminescence imaging, afterglow imaging detects slow release of photons from the chemical or energy defects stored by light pre-irradiation (Xu et al., 2016; Zhen et al., 2018; Lyu et al., 2019). The separation of light irradiation and signal collection circumvent the autofluorescence, inducing remarkable improvement of imaging sensitivity and SBR. Afterglow luminescence possesses extremely long lifetime, up to minutes to hours, which is long enough to implement time-resolved bioimaging without additional instrument. To achieve deep-tissue imaging, some afterglow nanoagents including inorganic and organic nanoparticles with NIR luminescence have been reported (Maldiney et al., 2011; Liu et al., 2013).

Inorganic persistent luminescence nanoparticles containing rare-earth metal ions have been explored as afterglow nanoagents for a long time. In the past 10 years, some advances have been made to prepare NIR emitting inorganic afterglow nanoparticles as highly sensitive tools for real-time bioimaging in living animals (Abdukayum et al., 2013; Li et al., 2014; Maldiney et al., 2014; Shi et al., 2015; Lécuyer et al., 2016; Ai et al., 2018). For instance, Han et al. employed an aqueous-phase reaction procedure to synthesize a water-soluble inorganic nanoparticle ZnGa_2_O_4_Cr_0.004_ with sub-10 nm in size and high NIR persistent luminescence intensity (Li et al., 2015). *In vivo* imaging result confirmed that afterglow nanoparticles had outstanding imaging ability with a SBR of 275. In addition, under the simulated deep-tissue environment created by covering a 1-cm-thick pork slab on living mouse, the renewable afterglow luminescence signal of the nanoparticle could be clearly observed. Imaging in NIR-II window (1,000–1,700 nm) possesses less light-tissue interactions (i.e., reduced light scattering) than those in NIR-I window, permitting imaging with higher SBR and sensitivity (Hong et al., 2014; Antaris et al., 2015; Sun et al., 2017; Huang et al., 2019; Tang et al., 2019a; Tian et al., 2019; Wang et al., 2019). Thus, to explore NIR-II afterglow agents, Zhang et al. synthesized a novel multifunctional nanoparticle mSiO_2_@Gd_3_Ga_5_O_12_:Cr^3+^,Nd^3+^ (mSiO2@GGO) (Shi et al., 2018). These mSiO2@GGO nanoparticles showed excellent afterglow luminescence in the first NIR window (745 nm) and second NIR window (1,067 nm). The NIR-I luminescence of the mSiO2@GGO could penetrate through the 2 cm-thickness tissue with a SBR of 5.5. To evaluate the NIR–II imaging ability of the mSiO2@GGO, the nanoparticles were subcutaneously injected into the abdomens of mice and a bright NIR–II luminescent signal was observed at the injection site. The result indicated that the mSiO2@GGO could be employed for deep-tissue imaging in NIR–II region.

**Figure 5:**
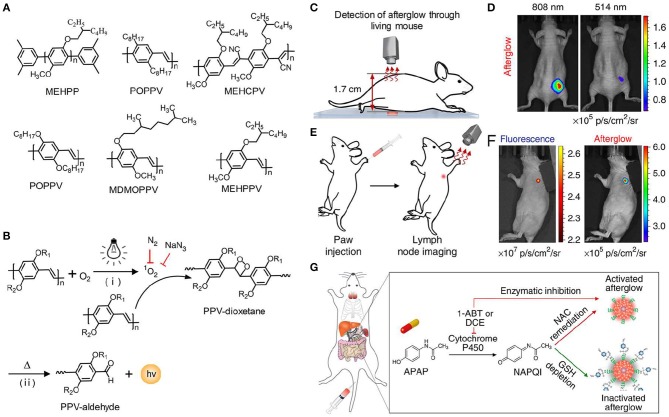
NIR afterglow luminescence imaging. **(A)** Chemical structures of MEHPP, POPPV, MEHCPV, BOPPV, MDMOPPV, and MEHPPV. **(B)** Illustration of the probable afterglow luminescence mechanism for PPV-based afterglow nanoparticles. **(C)** Illustration of tissue penetration experiment of SPN-NCBS through a living mouse (1.7 cm). **(D)** Representative afterglow images of SPN-NCBS solution through a living mouse with pre-irradiation of 808 or 514 nm laser. **(E)** Illustration of afterglow imaging experiment of a lymph node in living mice, after pre-irradiation with 808 nm laser for 1 min, the SPN-NCBS solution was stored in −20°C for 1 day and then was directly applied for lymph node imaging. **(F)** Representative afterglow (right) and fluorescence (left) images of lymph node in living mice. **(G)** Schematic illustration of SPN-NCBS with DNBS for detecting APAP-caused hepatotoxicity. The formation of N-acetylparaquinonimine (NAPQI) by metabolism of APAP with Cytochrome P450 can deplete GSH, leading to an inactivated afterglow. The antioxidant drug (N-acetyl-L-cysteine, NAC) and enzyme inhibitors (DCE or 1-ABT) remediate hepatotoxicity, causing an active afterglow (Reproduced with permission from Miao et al., 2017).

Although significant achievements in deep-tissue imaging have been made, inorganic afterglow nanoparticles may suffer from potential toxicity due to the existence of heavy metal ions (Toppari et al., 1996). Furthermore, the surface of inorganic nanoparticles is difficult to modify, thereby leading to the finite targeting ability. As an alternative, organic afterglow nanoparticles have attracted increasing interest due to their high biological safety, optical tunability, facile processability, and easy functionalization (An et al., 2015; Su et al., 2018). In 2015, Rao et al. reported the semiconducting polymer nanoparticles (SPNs) with afterglow luminescence that can last for an hour (Palner et al., 2015). However, the underlying mechanism of the afterglow phenomenon remained unrevealed. To resolve it, Pu et al. screened a series of semiconducting polymers and discovered that only phenylenevinylene (PPV)-based SPNs such as BOPPV, MDMOPPV, and MEHPPV had distinct afterglow luminescence, implying its essential role in the production of afterglow luminescence (Figure 5A; Miao et al., 2017). According to series of characterizations and analyses, the probable mechanism of afterglow luminescence for PPV-based SPNs was proposed as follows: the singlet oxygen (^1^O_2_) was generated by the light irradiation of PPVs and then oxidized the vinylene bond to form an unstable PPV-dioxetane intermediate, which could spontaneously decompose into PPV-aldehyde fragments along with photonic efflux (Figure 5B). To amplify the afterglow of SPNs and red-shift it into NIR region, NCBS, a NIR dye (778 nm) and ^1^O_2_ photosensitizer, was doped into SPN-MEHPPV to yield SPN-NCBS. The afterglow of SPN-NCBS could be increased by 11-fold under pre-irradiation at 808 nm compared to 514 nm due to the higher ^1^O_2_ generation ability of NCBS vs. MEHPPV. Due to the amplified brightness and lower background noise, the afterglow signal of SPN-NCBS could penetrate through a living mouse with a SBR of 237, which was 120 times higher than that of NIR fluorescence (Figures 5C,D). Then the SPN-NCBS was used for afterglow imaging of lymph nodes and tumors in living mice (Figure 5E). The afterglow of SPN-NCBS permitted more efficient and high-contrast imaging of lymph nodes and tumor relative to NIR fluorescence imaging, demonstrating its potential in guiding intraoperative surgical resection (Figure 5F). An overdose of drugs, take acetaminophen as an example, will induce oxidative stress after metabolization and in turn deplete antioxidants (i.e., biothiols) in body. Thus, imaging of biothiol levels can be a feasible way to evaluate drug-induced hepatotoxicity. To achieve this, a biothiol-activated afterglow probe was prepared for detecting drug-induced hepatotoxicity by introducing an electron-withdrawing quencher (2,4-dinitrophenylsulfonyl, DNBS) onto the surface of SPN-NCBS (Figure 5G). In the presence of biothiols, such as Cys, Hcy and GSH, DNBS was removed from the nanoprobe and then the electron transfer between the quencher and the afterglow moiety was restrained, leading to an activated afterglow. *In vivo* data proved that the probe permitted real-time afterglow imaging of drug-caused hepatotoxicity with an excellent SBR that is 25-fold higher than that of NIR fluorescence imaging.

Strong hydrophobic polymer required amphiphilic polymer to form a water-soluble nanoparticle by co-precipitation, thus leading to large size (34 nm) that could be unfavorable for efficient accumulation in tumor. To solve it, Pu et al. prepared an amphiphilic PPV polymer with grafted poly(ethylene glycol) that could self-assemble into smaller nanoparticles (24 nm) in aqueous solution (Xie et al., 2018). The nanoparticles were further doped with NCBS to form SPPVN-NCBS. Compared with SPN-NCBS, SPPVN-NCBS had smaller size, stronger NIR afterglow luminescence and higher accumulation in tumor. The NIR afterglow of SPPVN-NCBS could clearly detect 1 mm^3^ xenografted tumors and invisible peritoneal metastatic tumors.

**Figure 6:**
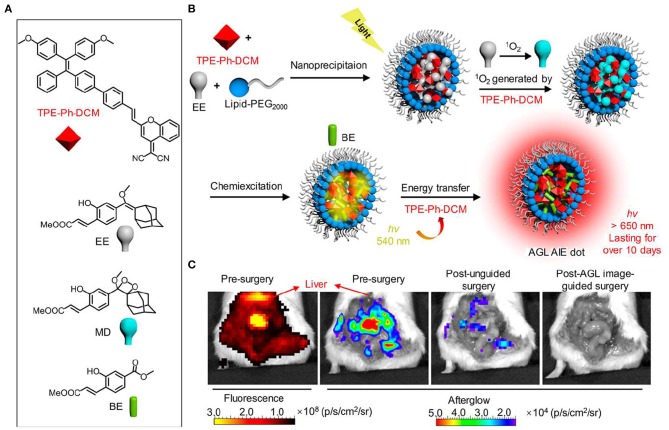
NIR AIE-based afterglow luminescence imaging. **(A)** Chemical structures of compound TPE-Ph-DCM, compound EE, MD, and BE. **(B)** Schematic illustration for NIR afterglow luminescence mechanism of AQL AIE dot. Under the white light pre-irradiation, TPE-Ph-DCM produced ^1^O_2_ that can oxidize compound EE to obtain compound MD. The slow degradation of compound MD leads to the formation of compound BE, and then the energy is transferred back to TPE-Ph-DCM to induce the NIR afterglow emission. **(C)** Fluorescence and afterglow images of the abdominal cavity of living mice with peritoneal carcinomatosis. The abdomen of mice was opened at 2 h after intravenous injection of AGL AIE dots (reproduced with permission from Ni et al., 2018b).

In a subsequent work, Pu et al. prepared a new library of afterglow luminescence agents by a generic approach that utilized an intraparticle cascade photoreaction of three main components (afterglow initiator, afterglow substrate and afterglow relay unit) (Jiang et al., 2019). By tuning the components, the afterglow emission could be adjusted from visible to NIR region. The representative NIR afterglow agents showed great imaging depth up to 5 cm, and permitted rapid detection of tumor in living mice with ultrahigh SBR (2,922 ± 121). Recently, Ding et al. reported the first aggregation-induced emission (AIE)-based NIR afterglow probe (AGL AIE dot) that could persist over 10 days (Figure 6; Ni et al., 2018b). This afterglow probe exhibited deep tissue penetration, high SBR, and ultrahigh signal ratios of tumor-to-reticuloendothelial system (RES) organs, thereby having the capability of image-guided cancer surgery in living mice. As shown in Figure 6C, the NIR afterglow imaging of AGL AIE dot could clearly differentiate the microtumors in peritoneal carcinomatosis-bearing mice, but the fluorescence imaging failed to do it. Moreover, under the guidance of the afterglow imaging, all the tiny tumor nodules could be nearly removed by surgery.

## Summary and Outlook

Self-luminescence including bioluminescence, chemiluminescence and afterglow have permitted imaging with higher sensitivity and deeper penetration depth than that obtained by fluorescence because it avoids the requirement of real-time light excitation and eliminates tissue autofluorescence. As NIR light possesses improved penetration depth than that obtained by visible light, many strategies via molecular- and nano-engineering approaches have been adopted to develop NIR self-luminescence. Stimulated by the merits, NIR self-luminescence imaging has been applied for *in vivo* detection of biological and pathological processes in living systems. With the development of new self-luminescence agents, the selectivity and sensitivity of bioluminescence, chemiluminescence and afterglow imaging tools have been dramatically improved.

Because bioluminescence, chemiluminescence and afterglow agents for deep-tissue optical imaging in living system is in the infancy, some challenges still need to be addressed. Firstly, the luminescence intensity of NIR self-luminescent agents is relatively low due to the limited efficiency to red-shift luminescence emission from visible to NIR window. Thus, it is necessary to explore new agents with evolving molecular- or nano-engineering strategy to obtain brighter luminescence. Secondly, compared with NIR-I light, NIR-II light has substantially lower light scattering, and thus increases the imaging sensitivity and tissue penetration depth (Jiang and Pu, 2018; Kenry et al., 2018). However, the second NIR (NIR-II, 1,000–1,700 nm) emitting self-luminescent agents have not been reported yet. We envision that new approaches will be developed to red-shift the emission of self-luminescent agent from NIR-I to NIR-II for advances of deep-tissue optical imaging. Thirdly, the majority of the reported NIR self-luminescent nanoagents have large size and are likely to retain in living organism for a long time, hence the long-term biosafety keeps unclear. To address this challenge, the nanoagents should be endowed with biodegradability and easily being cleared out by body. With the emergence of self-luminescent agents with high performance, it is confident that self-luminescence imaging will become a powerful tool in biology and clinical diagnosis of diseases.

## Author Contributions

QM and MG conceived this manuscript. QL, QM, JZ, and MG wrote and edited this manuscript.

### Conflict of Interest

The authors declare that the research was conducted in the absence of any commercial or financial relationships that could be construed as a potential conflict of interest.

## Abbreviations

SBR: signal-to-background ratio
NIR: near-infrared
SPECT: single-photon emission computed tomography
PET: positron emission tomography
MRI: magnetic resonance imaging
CT: computed tomography
PA: photoacoustic
Fluc: firefly luciferase
Gluc: *Gaussia* luciferase
Rluc: *Renilla* luciferase
Nluc: Nanoluc
BRET: bioluminescence resonance energy transfer
QDs: quantum dots
FRET: fluorescence resonance energy transfer
MEHPPV: poly [2-methoxy-5-((2-ethylhexyl)oxy)-p-phenylenevinylene]
NCBS: silicon 2,3-naphthalocyanine bis(trihexylsilyloxide)
cRGD: cyclic arginine-glycine-aspartic
ROS: reactive oxygen species
CRET: chemiluminescence resonance energy transfer
ONOO^−^: peroxynitrite
H_2_O_2_: hydrogen peroxide
CPPO: Bis-(2,4,5-trichloro-6-(pentyloxycarbonyl)phenyl) oxalate
PFODBT: poly(2,7-(9,9′-dioctylfluorene)-alt-4,7-bis(thiophen-2-yl)benzo-2,1,3-thiadiazole)
APAP: acetaminophen
RNS: reactive nitrogen species
TCPO: bis(2,4,6-trichlorophenyl) oxalate
HOMO: highest occupied molecular orbital
LUMO: lowest unoccupied molecular orbital
CIEEL: chemically initiated electron exchange luminescence
QY: quantum yield
Ce6: chlorin e6
MPO: myeloperoxidase
DCMC: dicyanomethylene-4H-chromene
LPS: lipopolysaccharide
FA: formaldehyde
SPNs: semiconducting polymer nanoparticles
PPV: phenylenevinylene
^1^O_2_: singlet oxygen
DNBS: 2,4-dinitrophenylsulfonyl
NAPQI: N-acetylparaquinonimine
AIE: aggregation-induced emission
RES: reticuloendothelial system.
